# Local anesthetic infusion pump for pain management following total knee arthroplasty: a meta-analysis

**DOI:** 10.1186/s12891-016-1382-3

**Published:** 2017-01-23

**Authors:** Yeying Zhang, Ming Lu, Cheng Chang

**Affiliations:** 1 Department of Anesthesiology, the Affiliated Hospital of Hangzhou Normal University, 126 Wenzhou Road, Hangzhou, Zhejiang 310015 China; 2 Department of Cardiology, Second Affiliated Hospital of Zhejiang Chinese Medical University, Hangzhou, Zhejiang 310005 China; 3 Department of anesthesiology, School of Medicine, Hangzhou Normal University, the affiliated Hospital of Hangzhou Normal University, 16 Xuelin St, Xiasha Higher Education Campus, Hangzhou, Zhejiang 310036 China

**Keywords:** Local anesthesia, Infusion pump, Total knee arthroplasty, Meta-analysis

## Abstract

**Background:**

We performed a systematic review and meta-analysis of randomized controlled trials (RCTs) were to evaluate the effect and safety of local anesthetic infusion pump versus placebo for pain management following total knee arthroplasty (TKA).

**Methods:**

In September 2016, a systematic computer-based search was conducted in the Pubmed, ISI Web of Knowledge, Embase, Cochrane Database of Systematic Reviews. Randomized controlled trials of patients prepared for primary TKA that compared local anesthetic infusion pump versus placebo for pain management following TKA were retrieved. The primary endpoint was the visual analogue scale (VAS) with rest or mobilization at 24, 48 and 72 h and morphine consumption at 24 and 48 h. The second outcomes are range of motion, length of hospital stay (LOS) and complications (infection, deep venous thrombosis (DVT), prolonged drainage and postoperative nausea and vomiting (PONV)).

**Results:**

Seven clinical studies with 587 patients were included and for meta-analysis. Local anesthetic infusion pump are associated with less pain scores with rest or mobilization at 24 and 48 h with significant difference. However, the difference was likely no clinical significance. There were no significant difference between the LOS, the occurrence of DVT, prolonged drainage and PONV. However, local anesthetic infusion pump may be associated with more infection.

**Conclusion:**

Based on the current meta-analysis, we found no evidence to support the routine use of local anesthetic infusion pump in the management of acute pain following TKA. More RCTs are still need to identify the pain control effects and optimal dose and speed of local anesthetic pain pump.

## Background

The number of primary total knee arthroplasty (TKA) procedures will be reached at 3.48 million in 2030 in the United States and the number will be as eight-fold to the year of 2005 [1]. TKA was associated with moderate to severe postoperative pain. It is reported that approximately 60% of patients have severe pain and 30% of patients have moderate pain after TKA [2]. The pain has follow specific characteristics: the occurrence in mobilization is higher than in rest; and pain intensity always peaking at 3 to 6 h after TKA and continuing for the following 72 h [3]. Achievement of pain relief after TKA is necessary and early pain control after TKA can increasing patients satisfaction and reducing the length of hospital stay [4, 5]. Several effective modalities are available, but each has its own drawbacks. These modalities including oral opiates, femoral nerve block (FNB) and local infiltration anesthesia [6, 7]. FNB may weaken the quadriceps strength and increase the occurrence of fall. Oral opiates will increase the complications such as the postoperative nausea and vomiting (PONV).

Local infiltration anesthesia (LIA) has been identified a successful and easy way to management postoperative pain, and promote early mobilization after TKA [8]. A meta-analysis indicated that LIA shows better pain control than FNB, which is considered to be the gold standard anesthesia method [9]. However, the anesthetic effects of LIA disappears within the first 24 h, attempts have been made to prolong the anesthetic effect using infusion pump for continuous local infiltration anesthesia (CLIA) [10]. There have been controversies about the effects of CLIA with infusion pump after TKA [11, 12]. Thus, we carried a systematic review and meta-analysis to re-assess the efficacy and safety of pain control of CLIA for pain control after TKA.

## Methods

### Search strategies

Two reviewers independently retrieved randomized controlled trials of CLIA for pain control in patients after TKA from PubMed, EMBASE, and the Cochrane Library. The search was last performed on August 23, 2016. There was no language restriction. The keywords and Mesh terms used in the search included “Arthroplasty, Replacement, Knee”[Mesh], “total knee arthroplasty”, “total knee replacement”, “TKA”, “TKR”, “Anesthesia, Local”[Mesh], “local infiltration anesthesia”. We selected local infiltration anesthesia for avoiding leave out relevant studies. The Boolean operators “AND” and “OR” were used to connect these terms. The bibliographies of all included studies and other relevant publications, including systematic reviews and meta-analyses, were traced to identify the missed relevant reports. Based on the titles and abstracts, 2 reviewers selected the potential eligible studies. And then the full text of the remaining articles was examined for eligibility.

### Inclusion and exclusion criteria

Inclusion criteria: Participants, patients with osteoarthritis or rheumatoid arthritis who prepared for primary TKA. Intervention and comparison-intervention group was continuous local infiltration anesthesia via infusion pump and the comparison group was placebo alone; Outcomes-The visual analogue scale (VAS) with rest or mobilization at 12, 24, 48 and 72 h. Study-Only randomized controlled trials were included in this study. Exclusion criteria: Participants, who underwent revision TKA; non-RCTs, comments, incomplete data for meta-analysis and letters.

### Data extraction and outcome measures

Two independent reviewers selected the eligible studies and extracted the following data from the included publication: the first author, year of publication, geographical location, number of patients, intervention and comparison, duration of the treatment, follow-up, patient characteristics, and study type. We contacted the first or the corresponding author for detailed study information. Any discrepancies between the 2 reviewers were resolved by an additional investigator.

The primary outcomes were the VAS with rest at 24, 48 and 72 h, VAS with mobilization at 24, 48 and 72 h and total morphine consumption. The secondary outcomes were range of motion (ROM) of knee at 6 month, length of hospital stay (LOS), and the complications (infection, deep venous thrombosis (DVT), prolonged drainage and postoperative nausea and vomiting (PONV)). We chose the longest time point to measure the range of motion of knee.

When standard deviations (SD) were not provided in a study, standard error of the mean (SEM) was transferred into SD. If necessary, the means, SD, or SEM were extracted from the available diagrams and tables by the software of “GetData Graph Digitizer”.

### Risk of bias assessment

The risk of bias tool was used to estimate the quality by using Review Manager, version 5.3 (The Nordic Cochrane Centre, The Cochrane Collaboration, Copenhagen, 2014). The sequence generation, allocation concealment, blinding of participants and personnel, blinding of outcome assessment, incomplete outcome data, selective outcome reporting, and other biases (baseline balance and fund) was measured and the risk of bias results was exported from Review Manager, version 5.3. Two authors independently assessed the quality of the studies, and disagreements were resolved via a discussion with a third author. And the level of agreement between the 2 reviewers was assessed with the Cohen kappa statistic and set the acceptable threshold value as 0.61 [13, 14].

### Statistical analysis

The meta-analysis was performed on the eligible data using Stata12.0 (Stata Corp, College Station, TX). The relative risk (RR) with 95% confidential intervals (CIs) was calculated for the dichotomous outcomes, and the mean difference (MD) with 95% CIs was calculated for the continuous outcomes. The I^2^ statistic was used to test the heterogeneity between studies. Heterogeneity was considered statistically significant if the I^2^ value was >50%. To assess the reliability of the results, a sensitivity analysis was performed by sequentially removing individual studies and recalculating the results. *P* < 0.05 was considered statistically significant and reported as a 2-sided test. Egger linear regression test and funnel plots would be implemented to estimate the publication bias.

## Results

### Search results and quality assessment

**Fig. 1:**
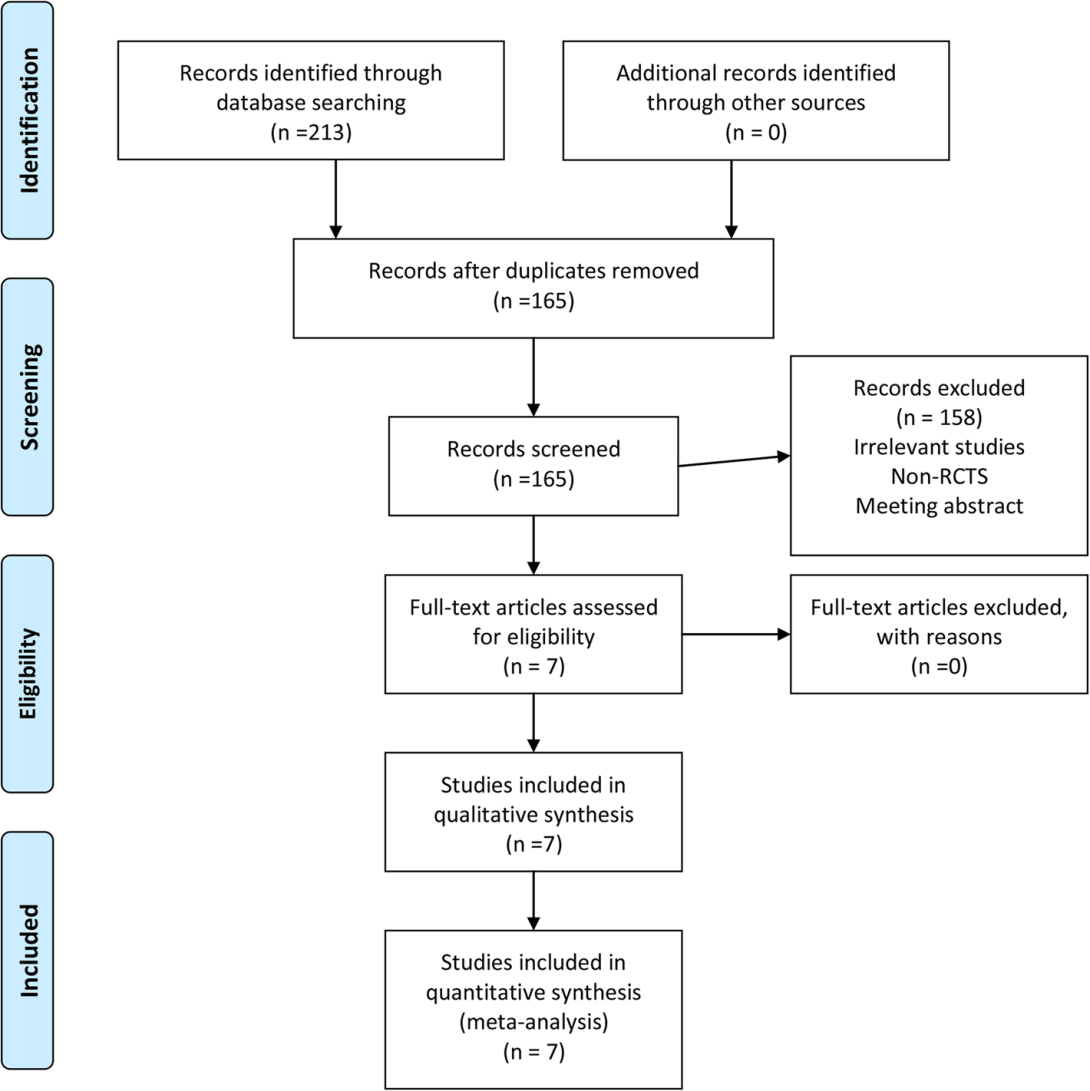
The flow diagram for the included studies

The initial search yielded 213 citations, of which, 48 duplicates were removed using Endnote software. After reading the titles and abstracts, 49 studies were excluded according to the inclusion criteria. Finally, 7 RCTs [10–18] with 587 TKAs were identified in our study (Fig. 1). The characteristics of the included studies are presented in Table 1. The included studies were published from the year of 2005 to 2015. The sample of patients ranged from14 to 97. The mean age of patients ranging from 63 to 71. And the volume of pain pump ranging from 100 to 300 ml. The delivery speed from 2 to 5 ml/h. Only one study did not state the delivery speed [10].

**Table 1:** The general characteristic for the included studies, 1 VAS with rest at 12 h, 2 VAS with rest at 24 h, 3 VAS with rest at 48 h, 4, VAS with rest at 72 h, 5, VAS with mobilization at 12 h, 6, VAS with mobilization at 24 h, 7, VAS with mobilization at 48 h, 8, VAS with mobilization at 72 h; 9, length of hospital stay; 10, morphine consumption at 24 h; 11, morphine consumption at 48 h; 12, the occurrence of infection; 13, nausea, 14; postoperative nausea and vomiting; 15, range of motion; 16, total morphine consumption

Reference	No. of patients		Male, %	Mean age, year	Intervention		Outcomes	Study design	Follow up
	CLIA	C			CLIA	C			
Nechleba 2005 [15]	14	16	63.3	65	200 cc 0.25% bupivacaine at 4.16 cc	200 cc Saline	2 3 11 12	RCT	6 week
Reeves 2009 [16]	31	30	41	69	240 cc 0.2% ropivacaine or 0.375% ropivacaine at 5 cc/h	240 cc Saline	2 3 6 7 8 11	RCT	2 day
Gomez-Cardero 2011	25	25	38	71	300 cc 0.2% ropivacaine at a speed of 5 cc/h	300 cc Saline	12	RCT	1 month
Zhang 2011 [12]	27	26	47.2	67	199 cc 2 mg/ml ropivacaine plus 2 ml 2 mg/ml ketorolac at 4 cc/h	199 cc saline	1 2 3 5 6 7 9 10 11 12 14	RCT	3 month
Goyal 2013 [10]	75	75	45.3	63	300 cc 0.5% bupivacaine	300 cc Saline	6 7 8 9 10 11 12	RCT	6 week
Williams 2013 [11]	26	25	41.2	67	0.5% bupivacaine at 2 cc/h	Saline	2, 3 9 13 9 10 14	RCT	1 y
Ali 2015 [18]	97	95	36.5	69	100 cc 7.5 mg/ml ropivacaine at 2 cc/h	100 cc Saline	2 3 4 7 10 11 12 13	RCT	3 m

**Fig. 2:**
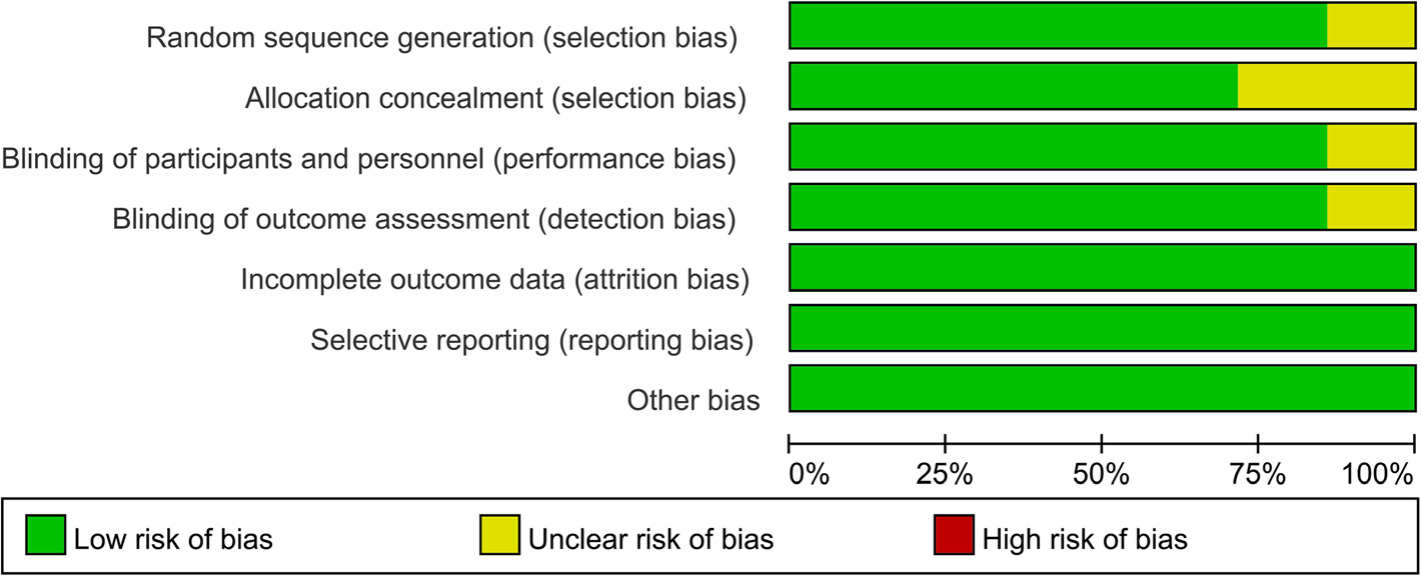
The risk of bias graph

**Fig. 3:**
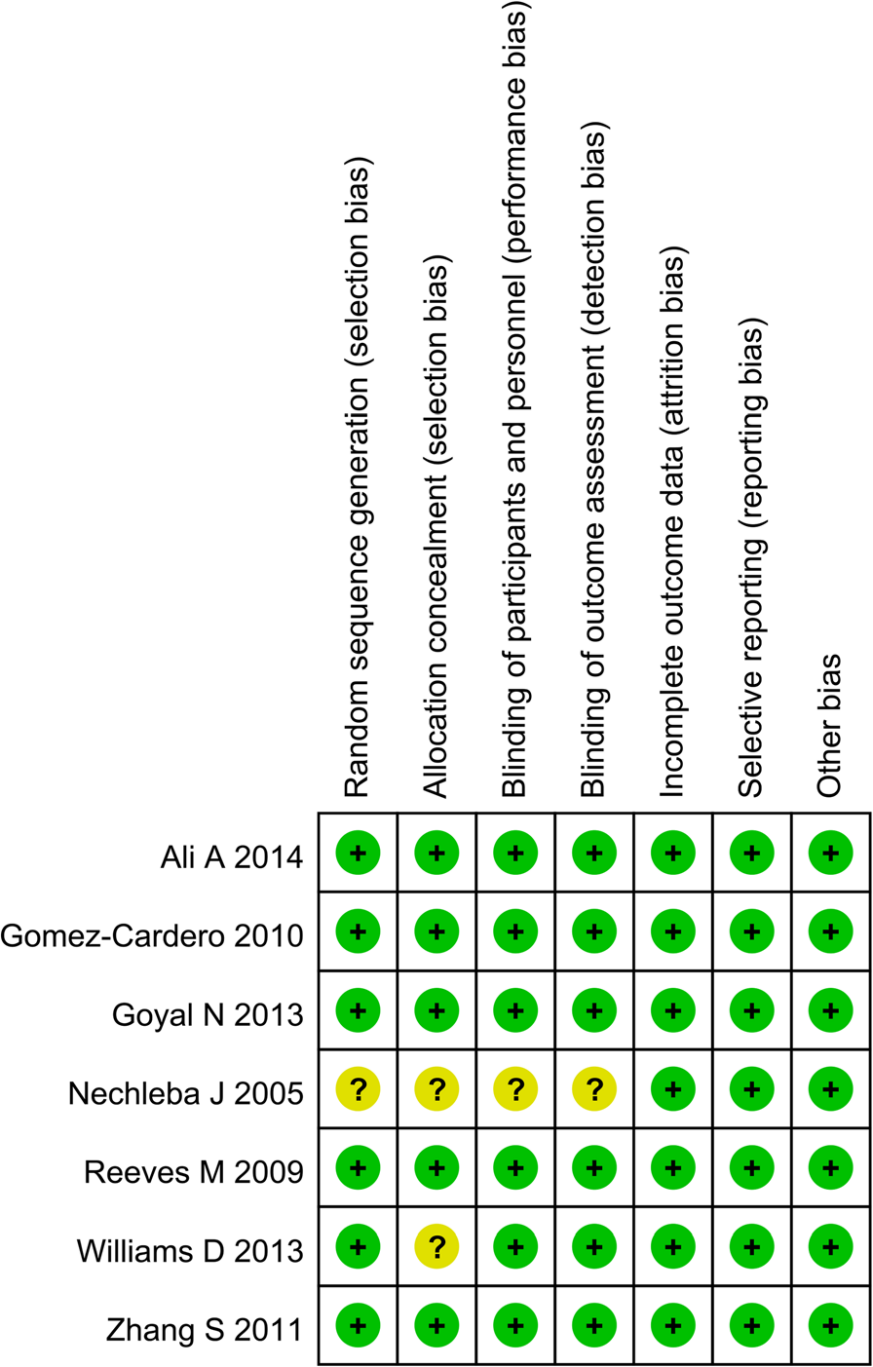
The risk of bias summary

The quality assessment results can be seen in Figs. 2 and 3. Only 1 study did not state the random sequence generation [15]. The other studies are all give appropriate random sequence generation. The allocation concealment are not clear in 2 studies [15, 16]. Blinding of participants and outcome assessment are unclear in 1 study [15]. The other bias are all with low bias. The agreement between the reviewers for risk of bias, based on kappa statistic, was 0.90.

### Results of the meta-analysis

#### VAS with rest at 24, 48 and 72 h

**Fig. 4:**
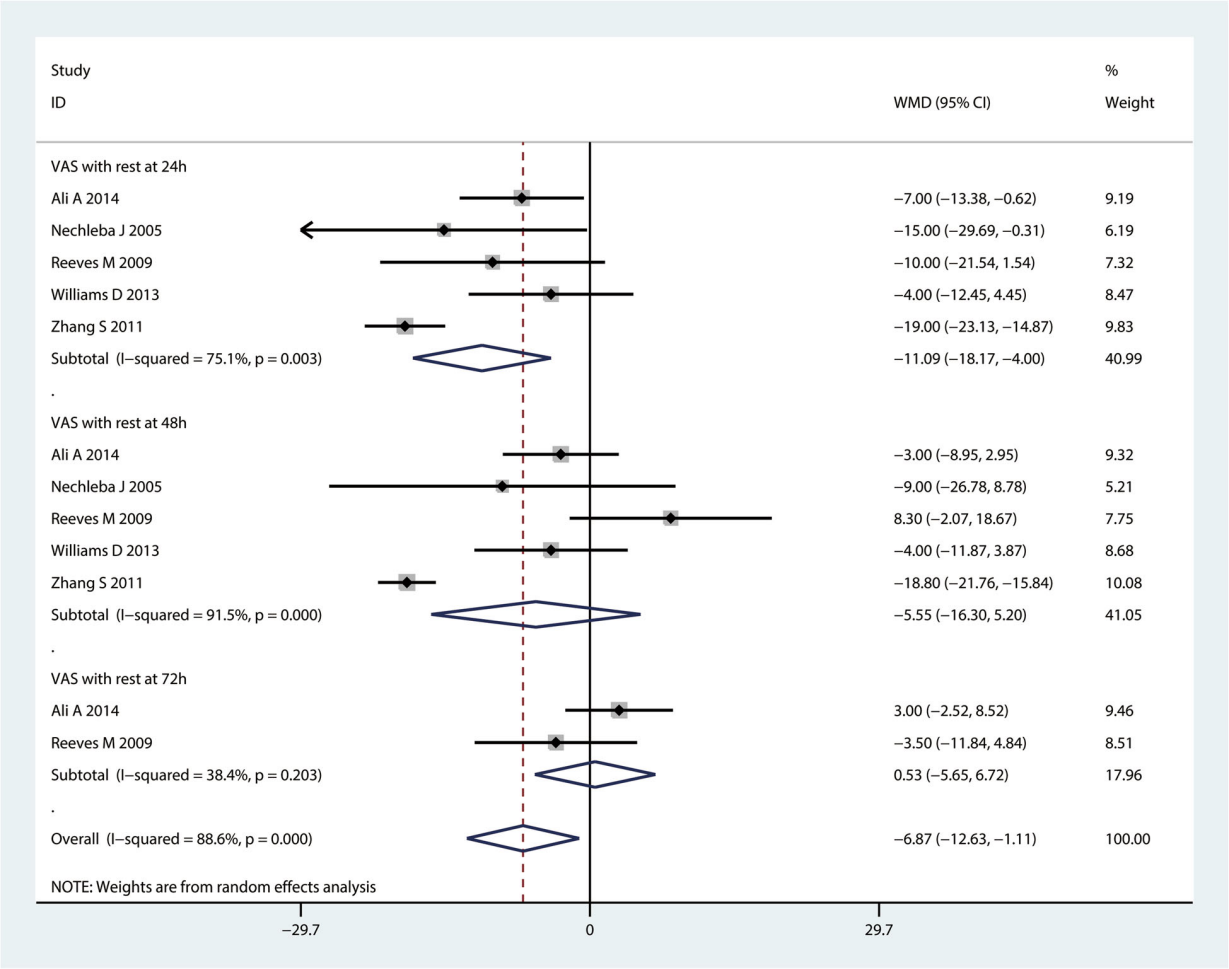
Forest plot comparing VAS with rest at 24, 48 and 72 h

**Fig. 5:**
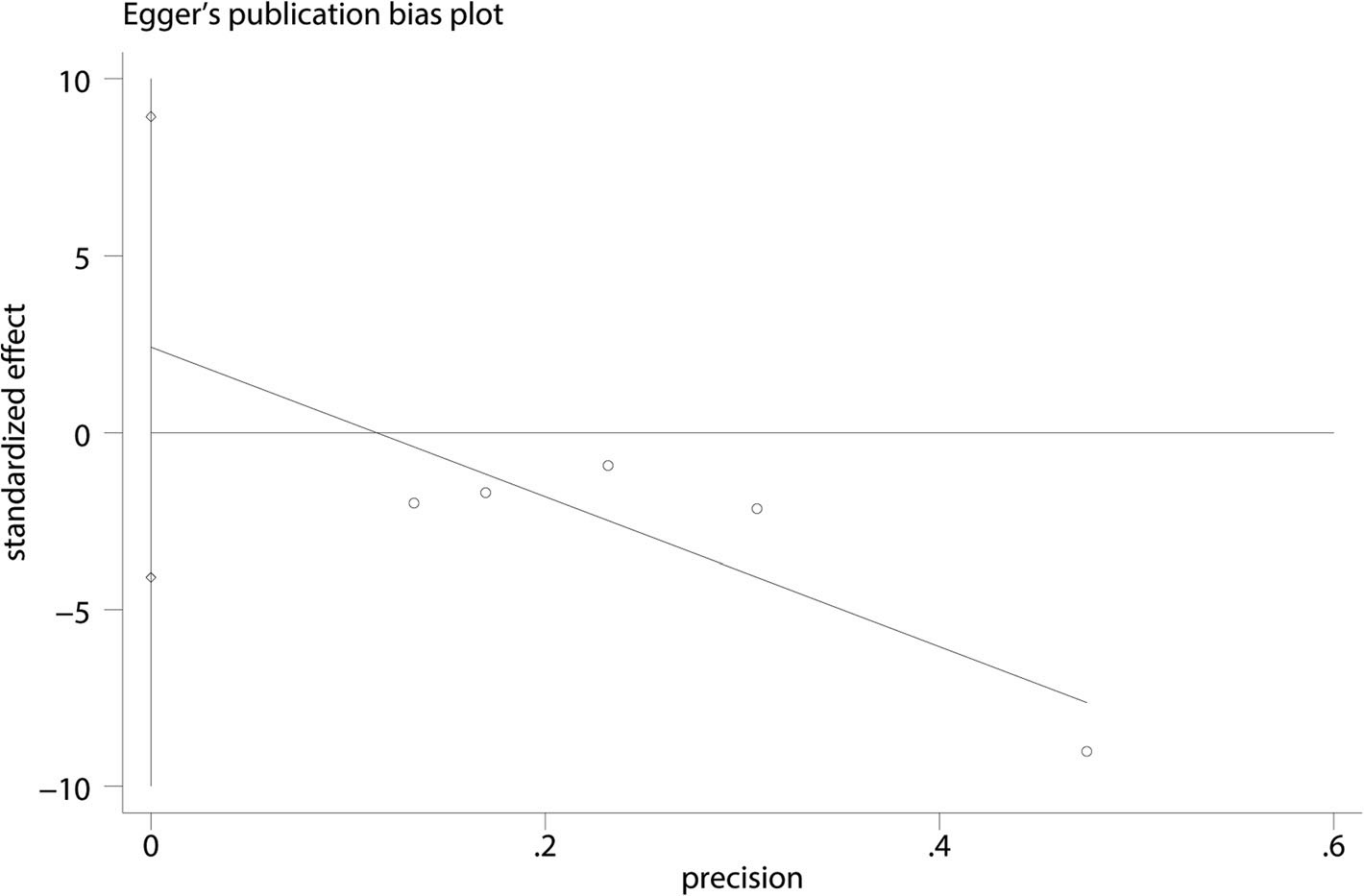
Egger’s test for publication bias for VAS with rest at 24 h

Five studies, including 384 patients, provided data for the VAS with rest at 24 h. The CLIA group was associated with a significant decrease in the VAS with rest at 24 h compared with the controls (MD = −11.09, 95% CI: −18.17–4.00, *P* = 0.002; I^2^ = 75.1%, Fig. 4). The data on the VAS with rest at 48 h and at 72 h were available from 5 studies and 2 studies respectively. There was no significant difference between the VAS with rest at 48 h (MD = −5.55, 95% CI: −16.30–5.20, *P* = 0.311; I^2^ = 91.5%, Fig. 4) and 72 h (MD = 0.53, 95% CI: −5.65–6.72, *P* = 0.866; I^2^ = 38.4%, Fig. 4) between CLIA group with control group. Egger’s test was performed to test the publication bias between the included studies. Results indicated that there was no publication bias between the studies (*P* = 1.000, Fig. 5).

#### VAS with mobilization at 24, 48 and 72 h

**Fig. 6:**
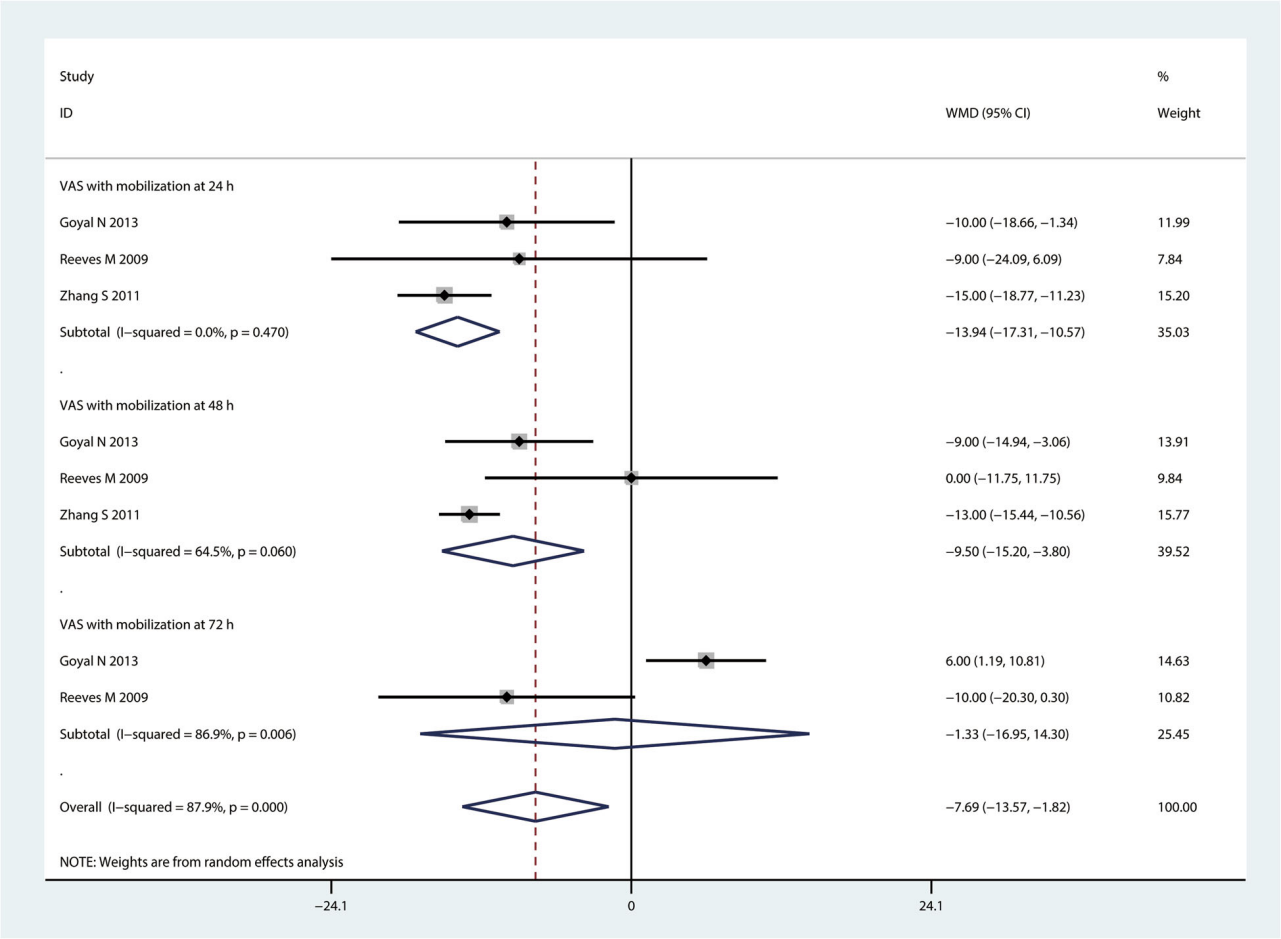
Forest plot comparing VAS with mobilization at 24, 48 and 72 h

Three studies (*n* = 263) contributed to the analysis of the VAS with mobilization at 24 h. The VAS with mobilization at 24 h was significantly decreased in the CLIA group compared with the controls (MD = −13.94, 95% CI: −17.31– −10.57, *P* = 0.000; I^2^ = 0.0%, Fig. 6). The data on the VAS with mobilization at 48 h were available in 3 studies, the pooled results indicated that CLIA can reduce VAS with mobilization at 48 h for a mean of 9.50 (95% CI: −15.20– −10.56, *P* = 0.001, Fig. 6). There was no significant difference between the VAS with mobilization at 72 h (MD = −1.33, 95% CI: −16.95–14.30, *P* = 0.868; I^2^ = 86.9%, Fig. 6).

#### Total morphine consumption

**Fig. 7:**
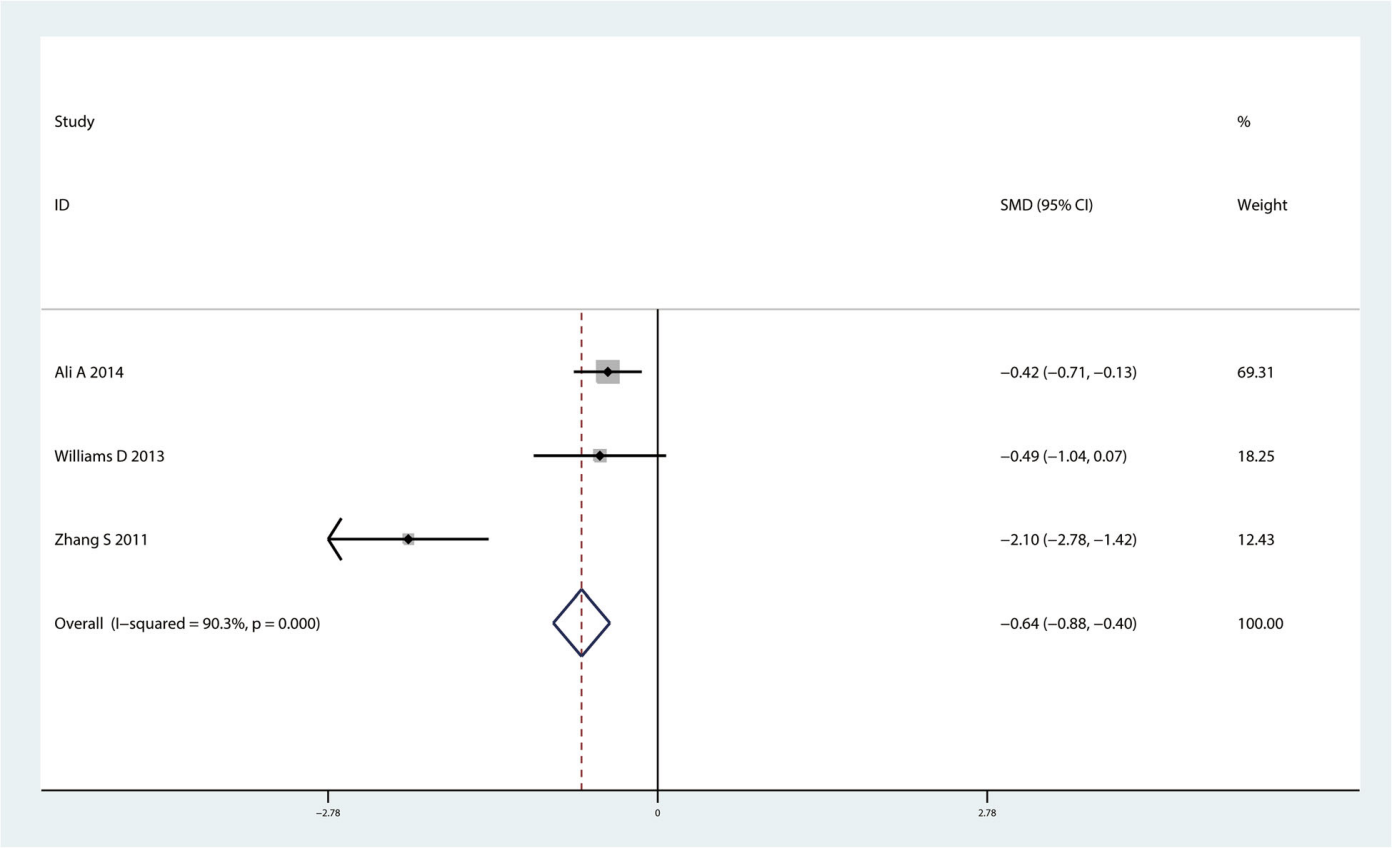
Forest plot comparing total morphine consumption between the two groups

In 3 studies (*n* = 296), provided the data for the total morphine consumption between the CLIA group with controls. Pooled results indicated that there was no significant difference between the morphine consumption between CLIA group with controls (SMD = −0.64, 95% CI: −0.88– −0.40, *P* = 0.000; I^2^ = 90.3%, Fig. 7).

#### ROM of knee at 6 month after TKA

**Fig. 8:**
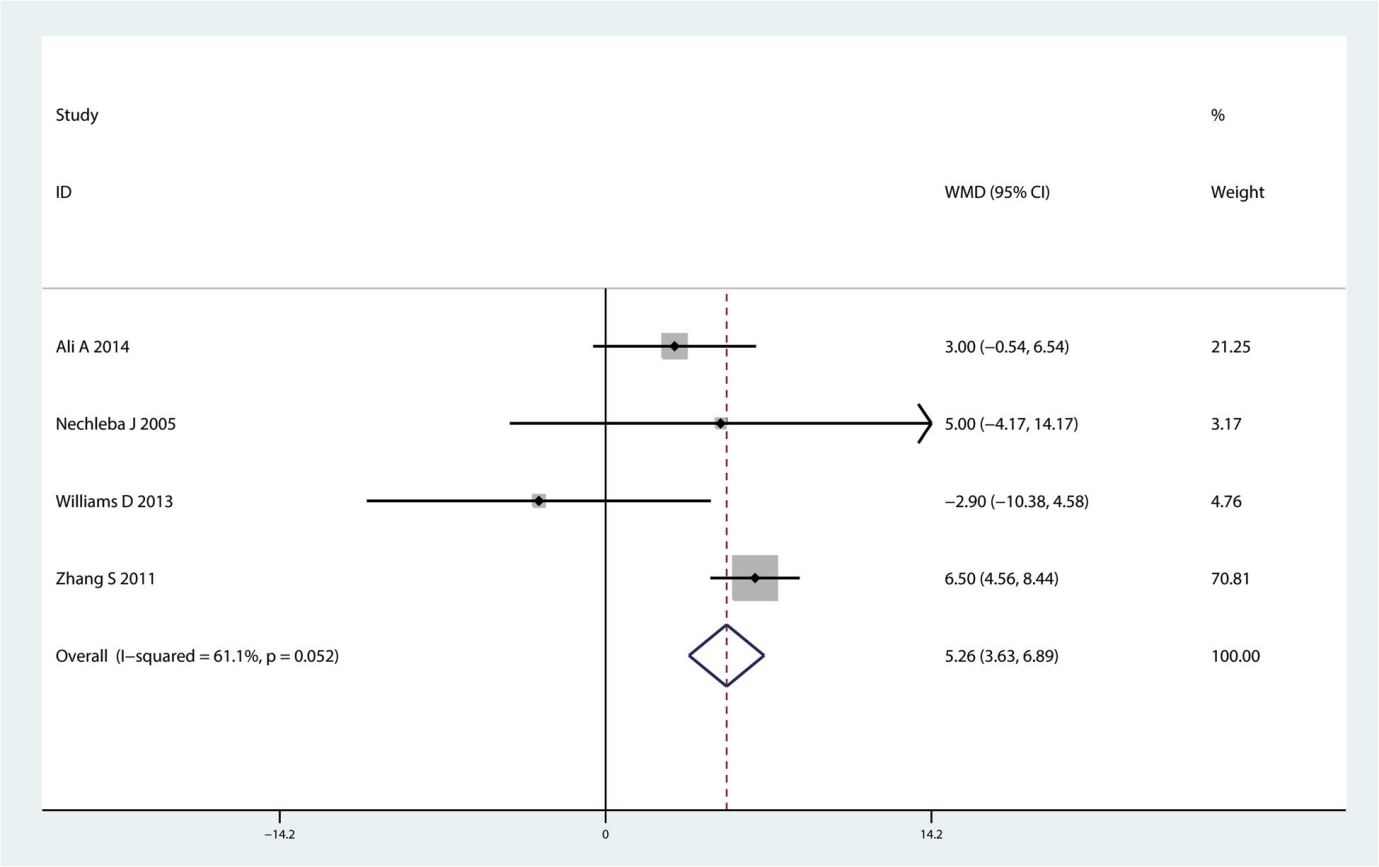
Forest plot comparing range of motion between the two groups

Four studies with 324 patients reported the relevant data of ROM of knee at 6 month after TKA. Pooled results indicated that, compared with control group, CLIA can increase the ROM of knee at 6 month after TKA (MD = 5.26, 95% CI: 3.63~6.89, *P* = 0.000, Fig. 8).

#### LOS

**Fig. 9:**
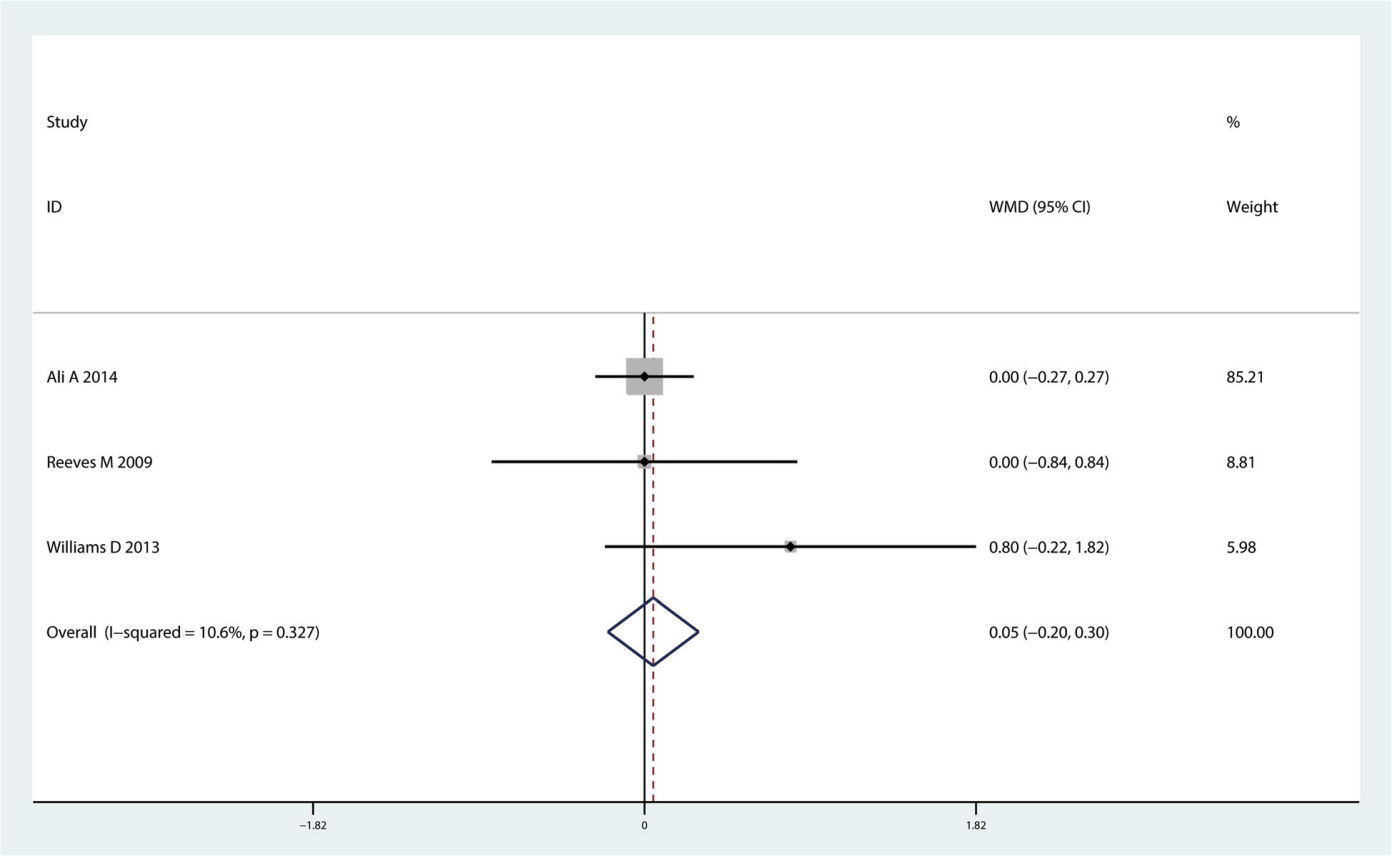
Forest plot comparing length of hospital stay between the two groups

Three studies (*n* = 411) contributed to the analysis of LOS. We observed similar LOS when comparing the CLIA group with the control group (MD = 0.05, 95% CI: −0.20~o.30, *P* = 0.086, Fig. 9).

#### Complications

**Fig. 10:**
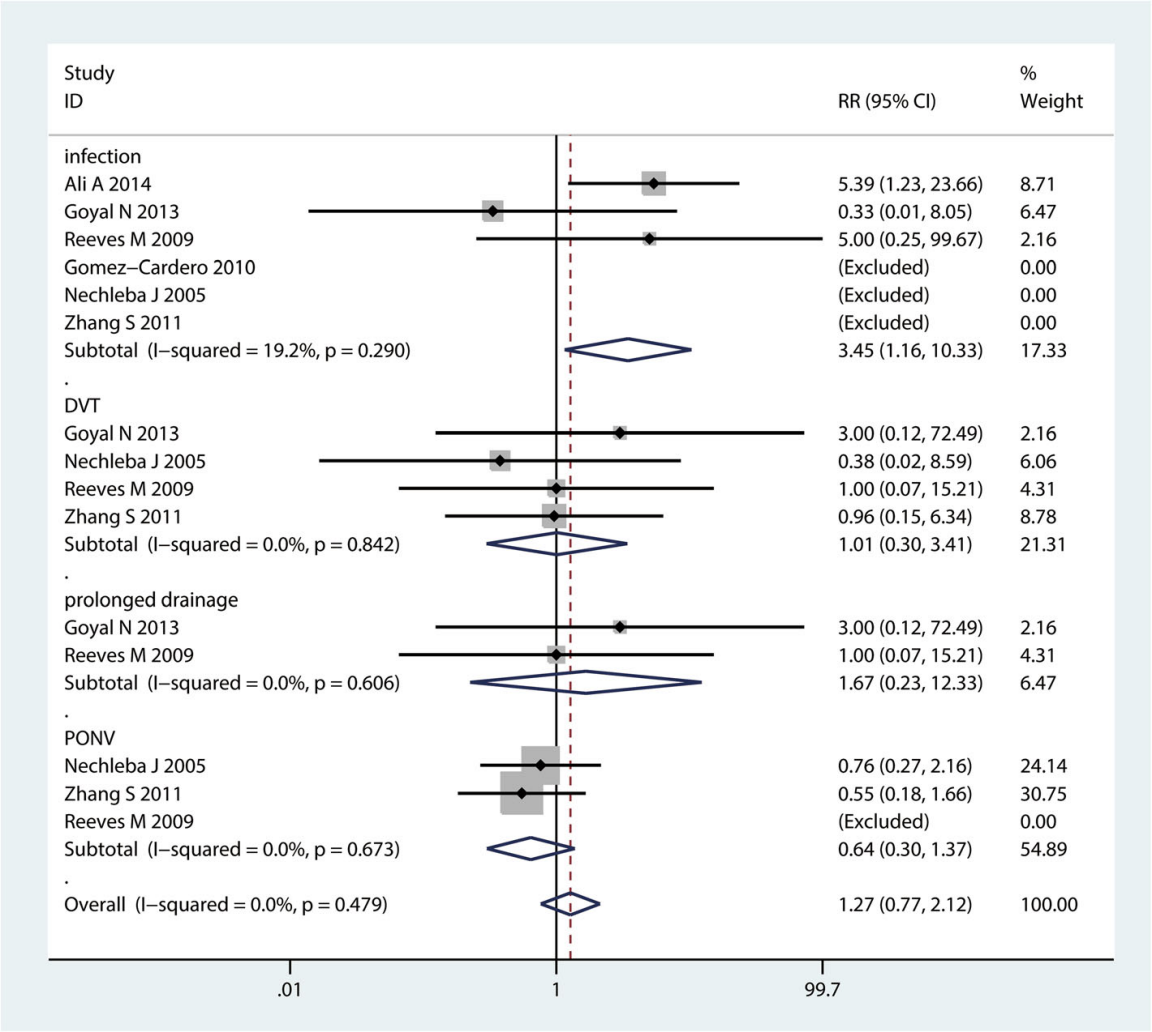
Forest plot comparing the complications between the two groups

Four common complications (infection, DVT, prolonged drainage and PONV) were compared between the included studies. Results indicated that there was no significant difference between the occurrence of DVT (RR = 1.01, 95% CI :0.30~3.41, *P* = 0.987, Fig. 10), prolonged drainage (RR = 1.67, 95% CI :0.23~12.33, *P* = 0.617, Fig. 10) and the occurrence of PONV (RR = 1.27, 95% CI :0.77~2.12, *P* = 0.255, Fig. 10). However CLIA increase the occurrence of infection (RR = 3.45, 95% CI :1.16~10.33, *P* = 0.027, Fig. 10).

#### Subgroup analysis

The results of VAS with rest has large heterogeneity between the included studies. Subgroup analysis was conducted according to the delivery speed (<2 cc/h or >2 cc/h) and the type of anesthesia drug (ropivacaine or bupivacaine). Pooled results indicated that local infiltration anesthesia with pain pump with a high speed (>2 cc/h) associated with less VAS with rest at 24 and 48 h than the low speed (<2 cc/h). And the local infiltration with ropivacaine is better than bupivacaine for reducing VAS with rest at 24 and 48 h (Table 2).

**Table 2:** The subgroup analysis for the VAS with rest or mobilization at 24 and 48 h

Outcome	Studies	Overall effects			Heterogeneity	
		Effect estimate	95% CI	*P* value	I^2^ (%)	*P* value
VAS with rest at 24 h						
low speed	2	−5.91	−11.00, −0.82	0.000	0.0	0.579
high speed	3	−17.78	−21.54, −14.02	<0.001	9.9	0.330
ropivacaine	3	−15.00	−18.32, −11.68	0.000	80.7	0.006
bupivacaine	2	−6.74	−14.06, −0.59	0.072	38.2	0.203
VAS with rest at 48 h						
low speed	2	−3.36	−8.11, 1.38	0.165	0.0	0.843
high speed	3	−17.79	−20.59, −14.98	0.000	56.5	0.100
ropivacaine	3	−15.22	−17.78, −12.66	0.000	91.5	0.000
bupivacaine	3	−4.82	−12.02, 2.38	0.189	0.0	0.614

## Discussion

This is the first systematic review and meta-analysis that compared local anesthetic infusion pump versus placebo for pain management following TKA. The pooled results indicated that administration with local anesthetic infusion pump can reduce pain intensity with mobilization and morphine consumption. The pain control and morphine consumption were likely of no clinical importance. There was no significant difference between the CLIA group versus placebo group in terms of LOS and complications such as PONV, DVT and prolonged drainage. However, the occurrence of infection in local anesthetic infusion pump group is higher than in placebo group. Though there was significant difference between pain score at 24 h with rest or mobilization, the clinical effects was limited. Thus, more RCTs for comparing local anesthetic infusion pump versus placebo after TKA are still need to identify the clinical value for pain control after TKA.

For this meta-analysis, we only included RCTs for meta-analysis, the quality of the included studies are all high and comparable. Only one study did not state the random sequence generation and the rest studies are all referenced the random sequence generation. All included studies all showed comparable characteristic and intent to treatment. The local infiltration drugs and drug delivery speed were different with each other. However, the half-time of all the local infiltration drugs were very nearly the same. The half-lives of bupivacaine was 3.5 h and ropivacaine was 2 to 6 h [19]. Thus, subgroup analysis was conducted to independent analysis the ropivacaine and bupivacaine for the VAS with rest at 24 and 48 h. Indirect comparison shown that the pain-sparing effects was better in ropivacaine group than in bupivacaine group. What’s more, the local infiltration anesthesia with a high speed is better than low speed group.

Pain control after TKA is always associated with the functional recovery and patients satisfaction [20]. An option that has gained popularity is peri-articular infiltration anesthesia or intra-articular anesthesia with pain pump for continuous infiltration. There is no consistent conclusion about the efficacy and safety of local anesthetic infusion pump for pain control after TKA [11, 12]. Williams et al. [11] conducted a RCT and found that pain score and morphine consumption are not significantly reduced when adding 48 h of 0.5% bupivacaine infiltration with pain pump. Ali et al. [18] also performed a RCT about CLIA with placebo for pain control after TKA and found that CLIA has no relevant clinical effect on VAS pain and does not affect LOS, morphine consumption but with a higher risk of wound-healing complications: deep infections. In this meta-analysis, the pain score was reduced 11.09 score with rest at 24 h and 13.94 score at 24 h with mobilization. This effect was likely of clinical importance. With time going on, the pain score was reduced 5.55 with rest at 48 h and 9.50 with mobilization at 48 h. The VAS with mobilization at 48 h was likely clinical importance. For VAS with rest or mobilization at 72 h, there were no clinical importance for these outcomes. Indeed, the attempts that administration with pain pump for prolong the analgesic effect still need for more studies to identify. The development of portable elastomeric infusion pumps can make the infusion of postoperative local anesthetic more easily available. However, if the pain control was limited in the first 24 h after TKA. The local infiltration anesthesia with long action bupivacaine can also reach the same effects. The costs and the operating time will be increased correspondingly. Zhang et al. [12] compared single-injection versus CLIA for pain control after TKA and found that CLIA provided prolonged superior analgesia and was associated with more functional recovery compared with single-injection local anesthesia. Wu et al. [21] conducted a meta-analysis to compare local anesthetic infusion pump for pain management following open inguinal hernia repair and found local anesthetic infusion pump was more efficacious for reducing postoperative pain than a placebo. The patients with different surgery type may suffer from variable pain intensity.

For morphine consumption, though CLIA can decrease the morphine consumption, however, only 0.64 mg was saved. The saving effects was found to be small of clinical effects. Thus, the effects of CLIA for morphine-saving is limited. For knee function, 4 studies with 324 patients perform range of motion of knee and pooled results indicated that CLIA can increase the ROM of knee for a mean of 5.26°. A total of 324 patients analyzed the ROM of the knee and thus further studies are needed to enhance the strength of the evidence presented. As for the complications, local anesthetic infusion pump are associated with more patients subjected to infection. Though the difference is statistically significant, the clinical significance worth deep research.

Our study has several limitations. First, the studies contain small samples, ranging from 14 to 75 patients per group, which might detract from the statistical power of the results. Second, several studies did not report the details of the generation and concealment of the allocation, and displayed other bias risks, such as the undetermined number of surgeons involved in the study. Finally, several of our primary and secondary outcomes were variably reported, thus potentially limiting the inferences based on our analysis.

## Conclusions

In conclusion, the results of our meta-analysis revealed that applying a local anesthetic infusion pump following TKA reduced postoperative pain compared to the placebo treatments during postoperative day 1 to day 2. However, the findings were based on a small body of evidence in which the methodological quality was not high. And the difference seems to be small of clinical effects. Based on the current evidence, we did not recommend routinely administration with local anesthetic infusion pump. Further research involving high quality RCTs might be warranted.

## Abbreviations

CIs: Confidential intervals
CLIA: Continuous local infiltration anesthesia
DVT: Deep venous thrombosis
FNB: Femoral nerve block
LIA: Local infiltration anesthesia
LOS: Length of hospital stay
MD: Mean difference
PONV: Postoperative nausea and vomiting
RCTs: Randomized controlled trials
RR: Relative risk
SD: Standard deviation
SEM: Standard error of the mean
TKA: Total knee arthroplasty
VAS: Visual analogue scale
